# Intracranial Hypertension Secondary to Venous Sinus Stenosis by Meningioma: A Case Illustration With Literature Review, Tips for Diagnosis and Management

**DOI:** 10.1002/ccr3.70357

**Published:** 2025-03-24

**Authors:** Sadegh Bagherzadeh, Milad Shafizadeh, Leila Bahari, Hamidreza Golestaneh

**Affiliations:** ^1^ Department of Neurosurgery Shariati Hospital, Tehran University of Medical Sciences Tehran Iran; ^2^ Sports Medicine Research Center Neuroscience Institute, Tehran University of Medical Sciences Tehran Iran; ^3^ Department of Physical Medicine and Rehabilitation School of Medicine, Tehran University of Medical Sciences Tehran Iran

**Keywords:** case report, pseudotumor, radiosurgery, shunt, stent

## Abstract

Intracranial hypertension due to venous sinus stenosis from meningioma is rare. Symptoms resemble pseudotumor cerebri, but patients are typically older and not obese. Tumors usually compress the superior sagittal, transverse, and sigmoid sinus. Ventriculoperitoneal shunt often leads to a dramatic response.

## Graphical abstract

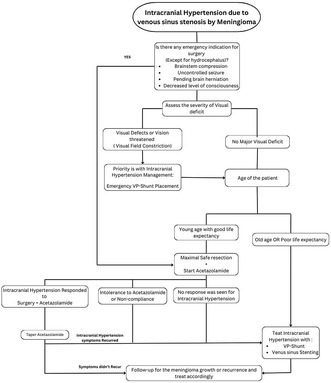

## 1 Introduction

Meningiomas are the most prevalent benign intracranial tumors in adults, accounting for one‐third of all intracranial tumors. These tumors can develop at any location along the meninges, which encases the central nervous system [1].

There are two types of intracranial hypertension: Primary and secondary, also known as idiopathic intracranial hypertension (IIH), and intracranial hypertension secondary to (a specific condition). Idiopathic intracranial hypertension (IIH), formerly referred to as pseudotumor cerebri, is characterized by elevated intracranial pressure (ICP) of unknown etiology. Before confirming a diagnosis of IIH, all other potential causes of elevated ICP must be thoroughly excluded [2].

Venous sinus outflow obstruction is present in 30% to 90% of patients with IIH. Increased pressure inside the skull may result from impaired blood flow due to blockage or significant narrowing of the posterior superior sagittal sinus, torcular herophili, sigmoid sinus, or dominant transverse sinus on one side [3, 4].

Meningioma is a brain tumor characterized by slow growth. Its mass effect can lead to increased ICP. While compression of the venous sinus resulting in venous hypertension and subsequent increase in ICP is rare, only a few cases have been reported [5, 6, 7, 8].

Venous sinus stenosis and its correlation with IIH are broadly discussed in the literature, but few reported cases of venous sinus stenosis and intracranial hypertension due to meningioma exist. We will present our case, review the existing literature, and provide an algorithm for managing this special subgroup of patients with meningioma.

## 2 Case History/Examination

### 2.1 History

A 53‐year‐old female was referred to the neurosurgery clinic by a neurologist. Her chief complaint was headaches with blurred vision since last year, which had progressed over the previous three months. She has been experiencing mild, throbbing headaches on both sides of her head, which worsen in the morning and when she strains. She has not experienced any nausea or vomiting. Additionally, she has been having brief episodes of temporary vision loss when standing up or straining for the past three months. She was diagnosed with migraines and has been taking propranolol 40 mg tablets twice a day and Sumatriptan‐Naproxen 80/500 mg tablets once daily for the past three months, but the treatment has not been effective. Aside from these symptoms, she has no other medical, drug, or surgical history, and her BMI is 23.7 kg/m^2^.

### 2.2 Physical Examinations

During the neurological examination, bilateral papilledema with Frisen Stage 4 was observed. In the confrontation test, her blind spot appeared larger than normal. There were no signs of nystagmus, and her cerebellar tests yielded normal results. All other cranial nerves were intact. Her muscle strength was normal (5/5 in the Medical Research Council (MRC) Scale for Muscle Strength), and both deep and superficial reflexes were within normal limits.

## 3 Methods (Differential Diagnosis, Investigations, and Treatment)

### 3.1 Diagnostic Assessment

**FIGURE 1:**
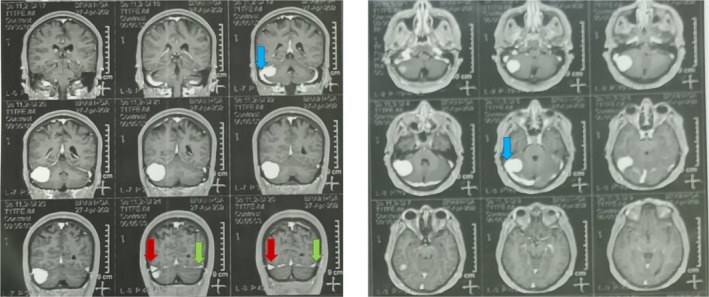
Preoperative brain MRI with Gad: There is an avidly enhancing lesion in the transverse and sigmoid sinus junction (blue arrows). The right transverse sinus (red arrow) is much more prominent than the left one (green arrow), indicating the right dominant venous sinus system.

**FIGURE 2:**
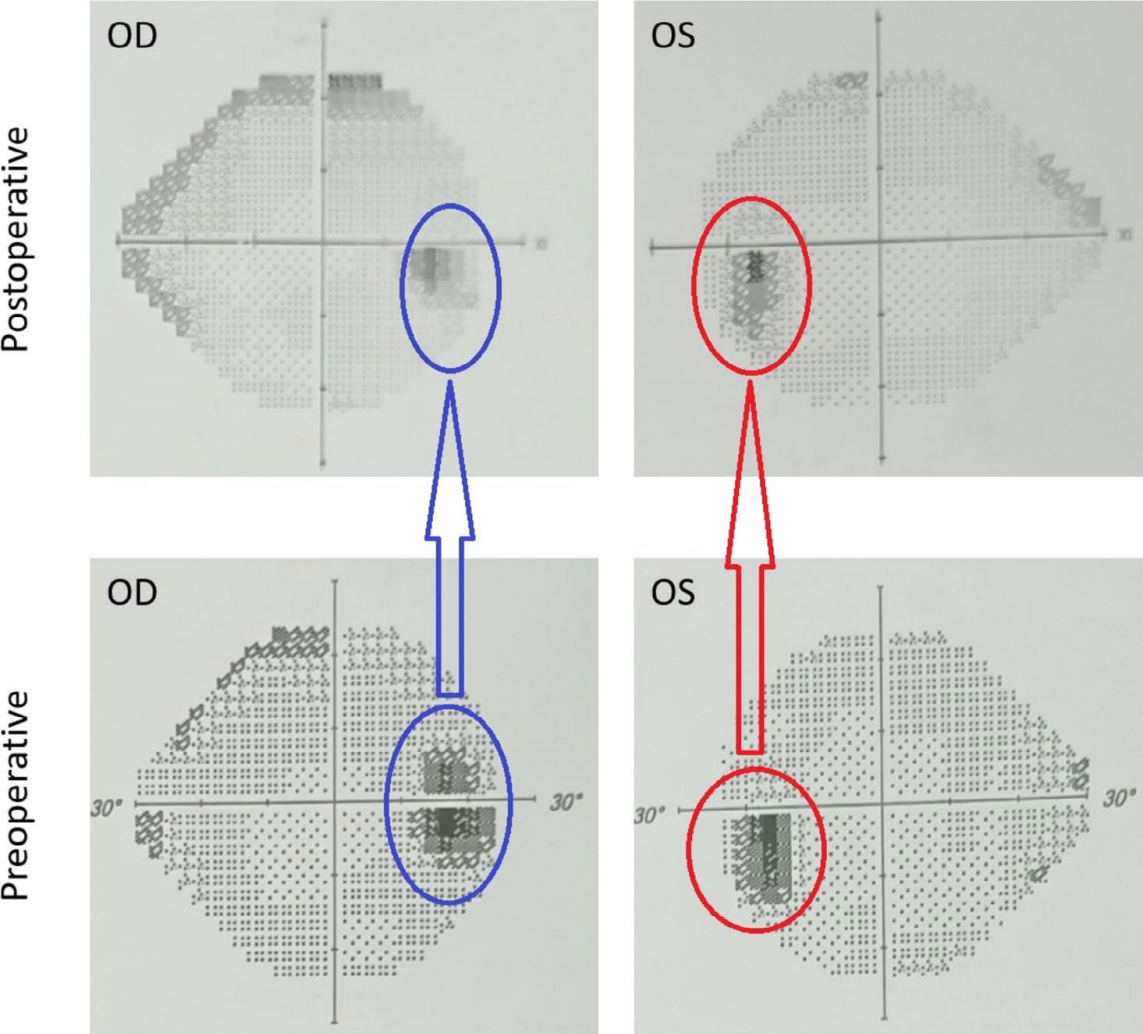
The patient's perimetry: The top row displays the postoperative perimetry, while the bottom row shows the preoperative results. OD stands for ocular dextra (right eye, blue arrow, and circles), and OS stands for ocular sinistra (left eye, red arrow, and circles). Comparing these two perimetries indicates that the size of the blind spot slightly increased before the surgery but returned to normal limits afterward.

Following IV contrast administration, the MRI scan identified an avidly enhancing mass located at the transverse‐sigmoid junction, resulting in venous sinus stenosis. The imaging also indicated that the patient's right venous sinus was predominant, as illustrated in Figure 1. As shown in Figure 2, visual field perimetry indicates that the patient's physiological blind spot is more prominent than normal. The perimetry results at the third‐month follow‐up are provided for comparison. Due to clinical signs and symptoms of the raised ICP, we performed a lumbar puncture in ICU, and the opening pressure was 36 cm H_2_O.

### 3.2 Therapeutic Interventions

We introduced the patient to our skull base surgeon's committee. Considering the size of the tumor and the patient's young age, we have elected to proceed with tumor resection. Following the procedure, we will closely monitor the patient's response to the resection using fundoscopy to assess intracranial hypertension.

We performed a right retro‐sigmoid craniotomy with neuro‐navigation to precisely locate the junction of the transverse and sigmoid sinuses (TSJ) and the tumor. After draining cerebrospinal fluid (CSF) from the cerebellomedullary cistern, we identified the tumor at the TSJ. We proceeded with its resection using a Fine‐tip CUSA (Cavitron Ultrasonic Surgical Aspirator). When we reached the TSJ, we intentionally left an eggshell layer of the tumor over the sinus (Simpson Grade IV) as the right transverse sinus was dominant, and damaging it could lead to a serious condition like Venous Infarction. We believed that by relieving the pressure of the tumor on the sinus, the venous flow might return to a normal state, and the patient's intracranial hypertension would likely improve. The patient's recovery in the postoperative period was uneventful. As per our ward's guidelines, we initiated prophylactic heparin (5000 units, subcutaneous, three times a day) 12 h after the surgery. The patient was discharged on the 3rd postoperative day with a prescription for Acetazolamide tablets (250 mg, oral, three times a day).

At the one‐month follow‐up visit, the patient reported no headaches and noted an improvement in her vision. Although papilledema was still present during the fundoscopy, the Frisen stage had improved to level 2 compared to before the surgery. She did experience side effects of Acetazolamide, such as dizziness, sleepiness, and tingling in her fingertips. As a result, we decided to reduce her Acetazolamide dosage gradually over 2 weeks.

During the 7th week following her surgery, she revisited the clinic and reported the recurrence of her symptoms. Although she was not experiencing any headaches, her vision had once again become blurred, and her fundoscopic examination revealed advanced swelling of the optic discs in both eyes (bilateral papilledema Frisen Stage 4). Taking into account the presence of papilledema, lack of improvement following tumor resection surgery, and the patient's intolerance to acetazolamide, we were presented with two options: either performing venous sinus stenting or placing a CSF diversion shunt. Access to endovascular neuro‐interventionists in our country is limited, and the cost of stenting is relatively high compared to income. As a result, we opted to place a programmable ventriculoperitoneal (VP) shunt through Kocher's point. The patient was discharged with the shunt in a medium‐pressure state on the second postoperative day.

## 4 Results (Outcome and Follow‐Up)

### 4.1 Pathology

The histopathological analysis illustrated the presence of a neoplasm with whorls and fascicles of elongated nuclei, inconspicuous nucleoli, and eosinophilic cytoplasm with an indistinct cytoplasmic border. There was an absence of apparent mitoses or necrosis. The pathological diagnosis identified the neoplasm as a transitional subtype meningioma, classified as WHO grade I.

### 4.2 Outcome and Follow‐Up

**FIGURE 3:**
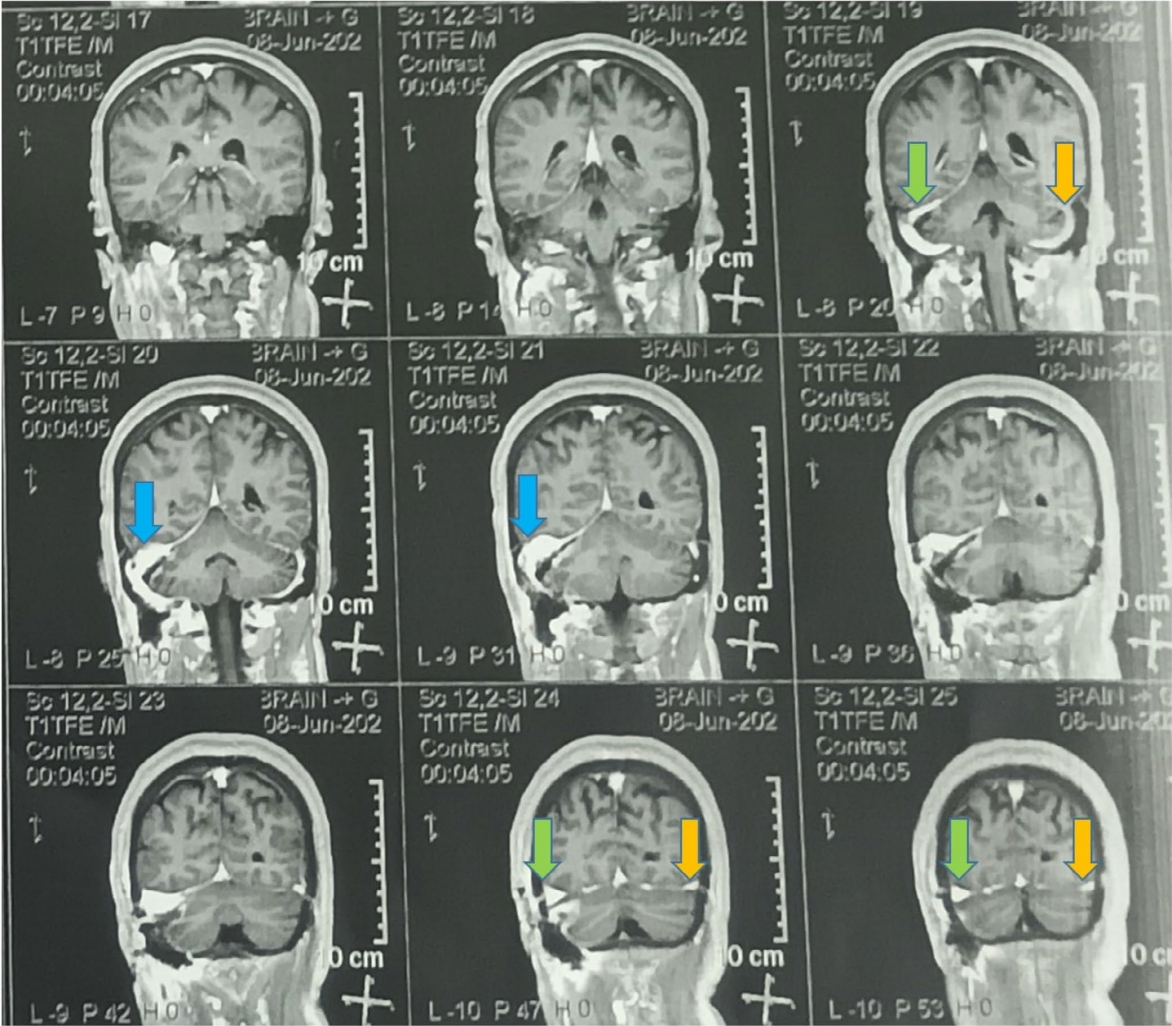
Postoperative brain MRI with Gad: The right transverse sinus (green arrow) is much more prominent than the left one (orange arrow), indicating the right dominant venous sinus system. The right sigmoid sinus (blue arrow) is dilated compared to the preoperative MRI.

The patient's first‐month follow‐up visit after VP‐shunt surgery showed that her shunt was functional and symptoms were completely gone. She had no headache and no blurred vision. Fundoscopic examination showed Frisen Stage 1. She was satisfied at the last follow‐up about 6 months postoperative, and her MRI didn't show residual tumor progression (Figure 3).

### 4.3 Similar Cases in Literature

We reviewed the literature and collected the reported cases of Meningioma plus intracranial Hypertension due to venous sinus stenosis in Table 1 [5, 9, 10, 11, 12, 13, 14, 15, 16]. The results of the descriptive analysis are shown in Table 2. The mean age was found to be 45.1 years, whereas, for IIH patients, it is reported to be 30 years [17]. Notably, a higher proportion of reported cases, similar to IIH patients, consisted of females, with a female‐to‐male ratio of 12 to 3. The average BMI of the patients was calculated to be 25.4. According to the World Health Organization, obesity is defined as a BMI exceeding 30 kg/m^2^, a characteristic often observed in IIH patients. Consequently, when presented with a patient exhibiting classic symptoms and signs of IIH but with an elevated age and a lower BMI, consideration of potential secondary causes is warranted.

**TABLE 1:** Reported cases of intracranial hypertension due to venous sinus compression by meningioma in the literature.

Author, year	Country	Age, gender	BMI	History	Presenting symptom	OP	Fundoscopy	Involved sinus	Tumor side	Meningioma location	Treatment	Headache outcome	Visual outcome
Index case	Iran	53, F	23.7	Migraine	Headache and TVO	High	Bilateral PE	Left TSJ	Left	Posterior petrous	Tumor resection, then VP shunt	Excellent	Improved
Healy, 2023 [5]	USA	42, F	22	Migraine	Headache and TVO	—	Mild bilateral PE	Left transverse	Left	Transverse sinus enplaque meningioma	—	—	—
Sumi, 2021 [9]	Japan	39, F	27.2	Neg.	Visual field abnormality	High	Bilateral PE	Right sigmoid	Right	Sigmoid sinus	Tumor resection	Excellent	Improved
Cheyuo, 2019 [10]	USA	17, M	—	Neg.	Headache and TVO	High	Bilateral PE	Right sigmoid	Right	Sigmoid sinus	Tumor resection + Radiosurgery	Excellent	Improved
Sharma, 2018 [11]	USA	32, F	24.8	Multiple meningioma	Headache and VI palsy	High	—	Posterior SSS	Right	Parasagittal	VP shunt, then gamma knife	Excellent	Improved
		40, F	31.3	Neg.	Headache and nausea	High	Bilateral PE	Right transverse	Right	Posterior petrous	VP shunt, then surgical resection	Excellent	Improved
		49, F	27.45	Neg.	Visual field abnormality	High	Bilateral PE	Posterior SSS	Right	Parasagittal	VP shunt, then gamma knife	Excellent	Improved
Shah, 2015 [12]	USA	43, F	—	Neg.	CSF rhinorrhea with mild headaches	High	—	Posterior SSS	Right	Parasagittal	VP shunt, then surgical resection	Excellent	—
		62, M	—	Neg.	Headaches and visual disturbances	High	—	Posterior SSS	Right	Occipital	Tumor resection, then LP shunt	Excellent	Improved
		43, M	—	Neg.	Headaches and visual disturbances	High	—	Posterior SSS	Right	Occipital parafalcine	Tumor resection	Excellent	Improved
		46, F	—	Neg.	Headaches and numbness in her right leg	High	—	Posterior SSS	Left	Parietal	Tumor resection	Excellent	—
Mariniello, 2013 [13]	Italy	28, F	—	Neg.	Headache and TVO	High	Bilateral PE	Right TSJ	Right	Transverse and sigmoid junction	Tumor resection	Excellent	Improved
Chausson, 2010 [14]	France	55, F	21	Neg.	Incidentally found PE	High	Bilateral PE	Left transverse	Left	Falcotentorial	Stenting	Excellent	Improved
Bouras, 2007 [15]	Greece	76, F	—	IHD and COPD	Visual field abnormality	High	Bilateral PE	Straight sinus	Right	Falcotentorial	LP shunt	Moderate	Improved
Truong, 1987 [16]	USA	52, F	—	Arterial HTN	Headache and TVO	High	Bilateral PE	Left transverse	Left	Tentorium	Tumor resection	—	—

Abbreviations: BMI, body mass index; COPD, chronic obstructive pulmonary disease; IHD, ischemic heart disease; LP, lumboperitoneal; OP, opening pressure; PE, papilledema; SSS, superior sagittal sinus; TSJ, transverse—sigmoid junction; TVO, transient visual obscuration; VI, abducens nerve; VP, ventriculoperitoneal.

**TABLE 2:** Results of descriptive analysis of reported cases of pseudo—tumor cerebri secondary to meningioma.

	Number (%)
*Demographic*	
Mean age (year)	45.1
Female to male	12:3
BMI (kg/m^2^)	25.4
*Involved sinus*	
Posterior SSS	6 (40)
Transverse	4 (26)
Sigmoid	2 (13)
TSJ	2 (13)
Straight	1 (8)
*Laterality*	
Right	10 (66)
Left	5 (33)
*Management*	
Only tumor resection	6 (40)
Shunt, then surgery or gamma knife	4 (26)
Tumor resection, then shunt	2 (13)
Only shunt	1 (8)
Only stent	1 (8)
Not reported	1 (8)

Abbreviations: BMI, body mass index; SSS, superior sagittal sinus; TSJ, transverse—sigmoid junction.

The most involved sinus was the posterior part of the superior sagittal sinus (40%), followed by transverse (26%), sigmoid (13%), TSJ (13%), and straight (8%). In 66% of patients, the involved sinus was on the right side; this can be explained by the fact that the right transverse sinus is dominant in about 57% of the normal population [18], so when the tumor compresses, this dominant sinus causes intracranial hypertension, but compression on the nondominant side may be asymptomatic.

In 40% of the patients, intracranial hypertension improved following tumor resection. Two patients who underwent tumor resection did not respond as expected, and a shunt was placed after surgery to manage intracranial hypertension. Twenty‐six percent of patients underwent shunt placement followed by surgery or Gamma Knife surgery. Additionally, there was a case in which symptoms were managed with venous sinus stent placement and another case with a lumboperitoneal shunt. As shown by previous studies, the VP shunt is more effective than the lumboperitoneal shunt (LP shunt) in IIH [19, 20].

## 5 Discussion

IIH refers to a condition characterized by elevated ICP in the absence of discernible intracranial pathological abnormalities, such as mass lesions or cerebral edema [2].

The exact cause of IIH is still unknown, but it likely involves the obstruction of the cerebral venous outflow [21]. The underlying pathogenesis of IIH is uncertain, with venous sinus stenosis being the most accepted cause [22]. This is because the most common finding in venographic studies in patients with IIH is stenosis of the transverse sinus [23], and it has been shown that increased brain volume may contribute to IIH pathogenesis by increasing extramural pressure on venous sinuses [24]. Although recently it has been proposed by Schartz et al. that the glymphatic system may have a role in IIH development, as IIH patients with transverse sinus stenosis had significantly lower glymphatic outflow than healthy controls [25]. Secondary causes may include venous thrombosis or a tumor. It is crucial to differentiate an intracranial tumor that compresses or invades the dural sinuses, such as a meningioma, from venous thrombosis or IIH [10].

The main principle of every surgery is to address the pathology with the lowest possible consequences for the patient. So, when we face patients like our case, we should consider different options and their consequences. Considerable caution should be exercised when contemplating surgery for lesions with unclear locations inside or outside the sinus or in cases where a tumor invades but does not entirely occlude the dominant transverse or sigmoid sinus, especially when outflow is heavily dependent on this sinus. Sacrificing the sinus under such circumstances can lead to severe complications, including hemorrhagic venous infarction, diffuse cerebral edema, seizures, or even fatality [26, 27].

**FIGURE 4:**
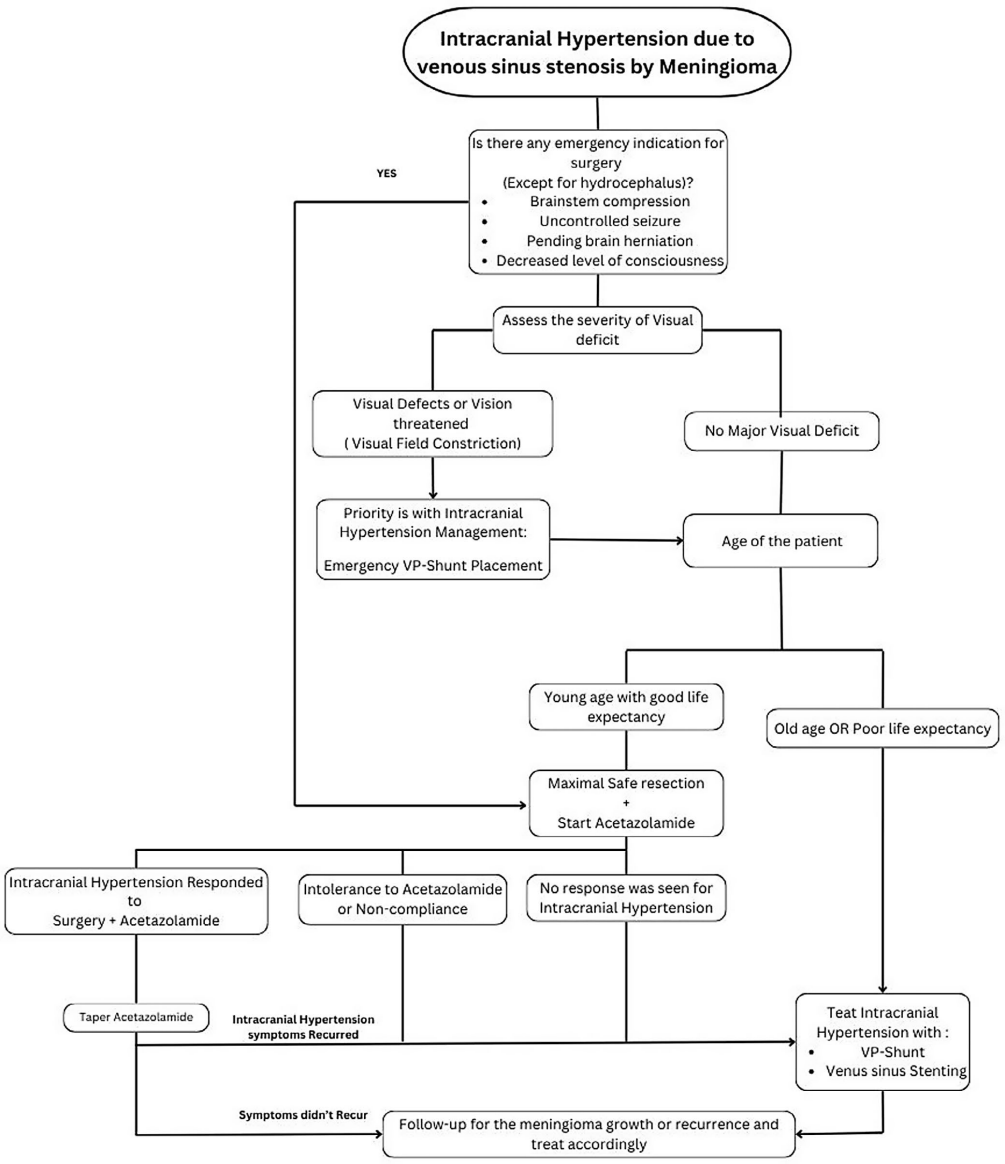
Proposed algorithm for management of intracranial hypertension due to venous sinus compression by meningioma.

When deciding on a management strategy, we should consider factors like the severity of visual impairment, the surgical resectability of the lesion, the dominancy of the involved sinus, the tumor growth rate, and the patient's age. Our proposed algorithm for managing concomitant intracranial hypertension due to venous sinus stenosis and meningioma is shown in Figure 4.

Suppose the patient had any indication for emergency surgery (except hydrocephalus). In that case, we propose to perform maximal safe resection (MSR) of the tumor, start Acetazolamide, and follow the intracranial response to surgery and Acetazolamide.

If the patient has no indication for emergency surgery, we will assess the severity of the visual defect; if the patient has a significant visual deficit, we proceed by placing the VP shunt. Otherwise, we will decide based on the patient's age and life expectancy. If the patient is young, we perform MSR, start Acetazolamide, and follow the patient. Still, if the patient is old or has a short life expectancy, we will treat intracranial hypertension with venous sinus stenting or VP shunt without tumor surgery. We recommend tumor resection for young patients for two main reasons. First, young patients have a longer life expectancy, allowing tumors more time to regrow. Second, evidence suggests that meningiomas involving major dural venous sinuses in a younger population are more likely to be World Health Organization grade II or III [28].

If the patients who had tumor resection surgery did not respond or had an intolerance to Acetazolamide, we will proceed with stenting or VP shunt. Nevertheless, all patients should be under surveillance for tumor growth or recurrence and treated according to existing guidelines for meningioma surveillance.

In our particular case, the patient's initial symptoms were indicative of IIH, with the exception of her not being obese or on any medication associated with IIH. Despite being treated for migraine, she did not respond to treatment and subsequently underwent a brain MRI. The MRI revealed the presence of a right TSJ meningioma causing compression over the sinus and resulting in sinus stenosis. Cerebral venous sinus thrombosis or stenosis can present with varying clinical symptoms, some resembling IIH. This underscores the importance of conducting venographic studies in patients with a clinical diagnosis of IIH [5].

A significant confounding factor that should be considered is the remnant tumor. The remnant tumor in our case and reported cases may contribute to the signs of the raised ICP apart from the compressive effect of the tumor on venous sinus and sinus stenosis.

## 6 Conclusion

Intracranial hypertension as a result of venous sinus stenosis due to meningioma is a rare occurrence. Individuals with this condition display typical symptoms and signs of pseudotumor cerebri but tend to be older (around 50 years) and often are not obese. It is more frequently associated with tumors compressing the posterior superior sagittal sinus, right transverse, and sigmoid sinus. When determining the appropriate management strategy, it is important to consider the severity of visual impairment, the feasibility of surgically removing the lesion, the dominance of the affected sinus, the tumor's growth rate, and the patient's age. Patients often exhibit a dramatic response to ventriculoperitoneal shunt.

## Author Contributions

**Sadegh Bagherzadeh:** conceptualization, data curation, investigation, visualization, writing – original draft, writing – review and editing. **Milad Shafizadeh:** conceptualization, supervision, writing – review and editing. **Leila Bahari:** conceptualization, methodology, writing – original draft. **Hamidreza Golestaneh:** visualization, writing – original draft.

## Ethics Statement

Our institution's ethical committee waived ethical approval for this case report because it was considered part of the usual patient care.

## Consent

The patient gave verbal and informed written consent to use his clinical data and images for publication in this case report; no identification of the patient's identity is present either in the manuscript or in the images.

## Conflicts of Interest

The authors declare no conflicts of interest.

## Data Availability

The article includes all the data regarding the presented case.
